# Multiaxial Fatigue Analysis of Connecting Bolt at High-Speed Train Axle Box under Structural Subharmonic Resonance

**DOI:** 10.3390/s23187962

**Published:** 2023-09-18

**Authors:** Yaqin Feng, Fansong Li, Kang Shu, Huanyun Dai

**Affiliations:** 1 State Key Laboratory of Rail Transit Vehicle System, Southwest Jiaotong University, Chengdu 610031, China; yaqinfeng@swjtu.edu.cn (Y.F.); lifansong@swjtu.edu.cn (F.L.); 2 College of Mechanical Engineering, Hunan Institute of Science and Technology, Yueyang 414000, China; 12022074@hnist.edu.cn

**Keywords:** dynamic stress, bolted joint, multiaxial fatigue, nonlinear vibration, axle box, high-speed train

## Abstract

Based on the dynamic characteristics of the axle box front cover of high-speed trains in the subharmonic resonance state, the nonlinear single-degree-of-freedom (SDOF) model was proved to be reasonable, and reasons for the ineffectiveness of the common prevention methods for preventing bolt failure were analyzed firstly. Then, dynamic stress of the bolt was simulated by innovatively adopting the linear method based on frequency response analysis. The stress simulation method was verified to be practical under the subharmonic resonance state by analyzing and comparing the experimental and numerical results of the bolted front cover. It was proved that the linear method was accurate enough to simulate the dynamic stress of bolts, which is of great engineering significance. In addition to the transverse resonance stress of bolts caused by drastic vertical vibration of the front cover, the tensile resonance stress at the root of the first engaged thread was too large to be neglected on account of the first-order bending modes of bolts. Next, equivalent stress amplitude of the multiaxial stresses was obtained by means of the octahedral shear stress criterion. Finally, fatigue life of bolts was predicted in terms of S-N curve suitable for bolt fatigue life analysis. It argued that the bolts were prone to multiaxial fatigue failure when the front cover was in subharmonic resonance for more than 26.8 h, and the fatigue life of bolts could be greatly improved when the wheel polygonization was eliminated by shortening the wheel reprofiling interval.

## 1. Introduction

### 1.1. Literature Survey

Since bolted joints are widely used in high-speed trains, their reliability is of great significance for the safety operation of vehicles. Loosening and fatigue failure of bolted joints are the two most important problems for vehicles that are subjected to vibration loading [1]. Abundant studies have demonstrated that self-loosening failure of bolts is mainly caused by dynamic transverse loading [2,3,4,5,6], and fatigue failure of bolts is usually caused by dynamic tensile loading [7,8,9,10]. However, transverse vibration can cause fatigue of bolted joints as well. Hashimura [11] proved that the loosening-fatigue life of bolted joints did not depend on the initial clamping force, but it significantly depended on the transverse vibration force. Minguez [12] reported that there was bending as well as shearing of bolted joints because the load transfer path was not symmetric for single lap joints. Increasing bolt tightening torque did not have any effect on the fatigue life for this type of joint because the main cause for fatigue failure was the strong bending moment caused by distortion. Benhaddou [13] and Guo [14] demonstrated that it was easy to generate stress concentration in the bolt hole, and increasing the preload of bolt could alleviate the stress concentration and transfer the cracking position. Wang [9] studied the fatigue behavior of stainless steel bolts under transverse loading and regarded the relative displacements of clamped parts as the deformations of bolts given the rigid clamped parts. Due to the high notch effect, bolts are more prone to fatigue failure than nuts or clamped parts; thus, it is of significance to study the stress state of bolts. However, the above studies mainly discussed the fatigue behavior of clamped parts rather than the transverse capacity of bolts itself. Traditionally, axial load of bolts can be monitored accurately in real time by means of strain gauged bush [15,16], smart washer [17,18], as well as embedding the sensor into the bolt shank [19]. Through the transverse dynamic load test of bolted joints, researchers found that cracks at the root of the first bolt thread could be observed after about 10^4^–10^6^ cycles when the loosening of bolts occurred [11]. But the transverse stress of bolts was less experimentally measured due to the limitation of measuring techniques. Although stress analysis of bolted joints is feasible by FEM [6,9], it is difficult to simulate the complex excitations of the actual operating state, which would result in a large difference between the calculated stress and actual stress. Especially when the structure is in a nonlinear vibration state, it cannot be excited in commercial FEA software such as ABAQUS, so the stress simulation results are not reliable. In short, it is not suitable to simulate the multiaxial stress of bolts under complex operation states by FEM.

Polygonal wear of wheel tread occurs frequently in Chinese railways, which can induce drastic vibration or even resonance and cause fatigue failure of critical structures; it is particularly dangerous for the structures fixed by bolts when the bolt axis is perpendicular to the structural vibration direction. Vibration characteristics and fatigue life under excitation of wheel polygonization were studied a lot for critical structures, such as gearbox housing [20], bearings [21] and lifeguards [22,23], etc.; the connecting bolts are usually subjected to multiaxial dynamic stresses in the real operation condition of high-speed trains. In the past few decades, the issue of damage mechanisms and stress combination of multiaxial loading have been brought to extensive attention, and significant research progress has been made by means of theoretical research and numerical analysis [24,25,26,27]. However, multiaxial fatigue life of connecting bolts was rarely studied because the dynamic stress of bolt threads is difficult to measure. Although metallographic analysis can qualitatively analyze material fatigue failure, the quantitative analysis cannot be realized because the cyclic stress level of the structure is unable to be obtained [28,29]. Consequently, it is of great importance to find a suitable dynamic stress calculation method for bolts.

In recent years, scholars have proposed some novel theoretical models for fatigue life prediction [30,31]. Although theoretical models can yield accurate solutions provided that accurate models can be built, it is difficult for structures under complex loads. There are several dynamic stress simulation methods combining experimental and numerical analysis suitable for bolt dynamic stress simulation. The quasi-static superposition method and the modal superposition method are two common methods in structural dynamic stress simulation. The quasi-static superposition method [32,33] is widely used during the design stage, but this method is not suitable for resonance stress simulation. As for the modal superposition method, the main idea is to obtain the load-stress TF (Transfer Function) of structure, and the external loads are often processed as PSD (Power Spectral Density) in prior research cases [32,34,35,36]; then, the stress PSD can be calculated by multiplying the load PSD by the load-stress TF. Although the modal superposition method is suitable for dynamic stress simulation under resonance conditions, the phase information of the load PSD and TF is ignored. In fact, the effect of the phase between multiaxial excitations on the stress response is so significant that it cannot be ignored; fortunately, the modal superposition method based on FFT of multiaxial excitations and load-stress FRFs takes into account the phase information of multiaxial excitations and was chosen as the bolt dynamic stress simulation method [22].

To simulate the dynamic stress of the bolt in resonance states of structures, the dynamic characteristics of the connected structure must be experimentally analyzed; therefore, processing of the experimental signal is also critical. Because the precision of the fast Fourier transform (FFT) and short-time Fourier transform (STFT) depend on the kind and size of window function, frequency leakage is easy to occur. To avoid leakage, the Hilbert-Huang transform (HHT) was introduced based on an algorithm called empirical mode decomposition (EMD) [37], which is widely used today to recursively decompose a signal into different modes of unknown but separate spectral bands, especially appropriate for nonstationary and nonlinear signals. However, EMD is known for limitations like modal aliasing and edge effects; thus, the algorithm was further improved with the EEMD [38] and CEEMD [39] methods. These methods removed the noise by adding a uniform white noise to the original signal; although mode aliasing can be avoided, amplitude of white noise has great influence on the calculation accuracy. With further improvement, the variational mode decomposition (VMD) method was proposed and proved to be effective in avoiding the shortcomings of the previous methods [40].

### 1.2. Research Background and Method

**Figure 1:**
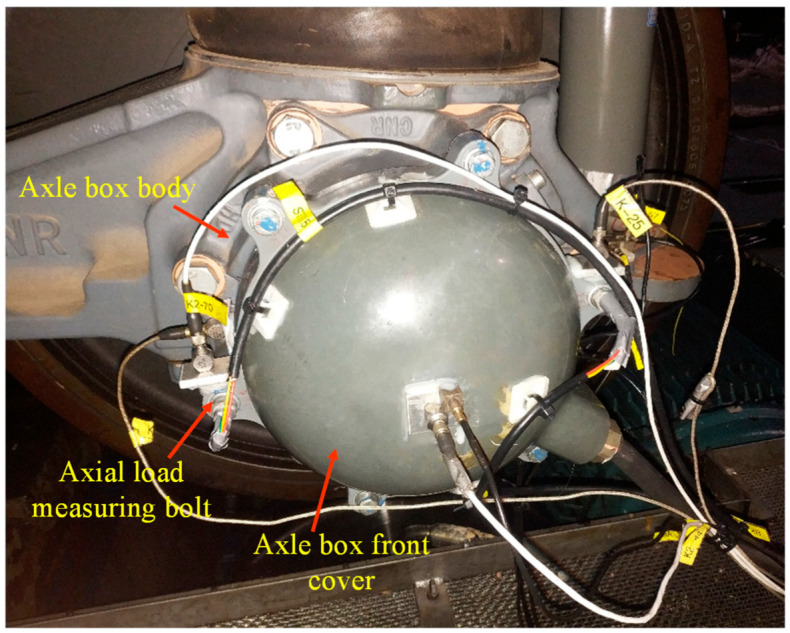
Structure of the axle box of high-speed train.

As a critical component in high-speed trains, the axle box is mainly composed of the axle box body and axle box front cover; the front cover is connected to the axle box body by six M8 × 40 steel bolts with strength grade of 8.8, as shown in Figure 1. After the high-speed train ran for a period of time on a certain railway line, the connecting bolts would loosen even fracture from time to time, but these failed bolts were theoretically far from reaching the design service life. To solve the problem, the failed bolt was replaced by a new bolt and some prevention methods were used to improve the service life of the connecting bolt, such as adopting a spring washer, replacing the lubricating oil with grease or increasing the preload; these methods are called common prevention methods in this study. However, the new connecting bolts still failed quickly, which obviously greatly threatened the operation safety of the high-speed train; only when the failure mechanism of the connecting bolt is found can the intractable problem be solved.

It was proved that the axle box front cover was in subharmonic resonance of order 1/2 under excitation of 20th-order wheel polygonization, and the resonance caused greater transverse stress of the bolted joint [41]. In this study, an effective method based on numerical and experimental analysis was proposed for multiaxial stress calculation of bolt threads. Firstly, effectiveness of the common prevention methods under structural resonance was assessed based on the nonlinear vibration response of the front cover. Next, feasibility of the method was proved in terms of the axial and transverse stiffness of bolted joints because of the existence of nonlinear characteristics of bolted joints and the first application of this linear stress simulation method on bolts. Then, multiaxial dynamic stresses at the root of the first engaged bolt thread were simulated, the equivalent stress was obtained by the octahedral shear stress criterion and adopted to assess the bolt fatigue life. Finally, a suggestion to improve the fatigue life of bolts was put forward.

## 2. Assessment for Effectiveness of Common Prevention Method

In this section, firstly, dynamic behavior of the front cover was analyzed according to results of the bench test under 20th-order wheel polygonization. Then, a nonlinear model of the front cover was built by a single degree of freedom (SDOF) modeling strategy, and the feasibility was verified. Finally, effectiveness of common prevention methods was assessed based on the nonlinear vibration response of the front cover.

### 2.1. Dynamic Characteristics of the Bolted Front Cover under Condition of Wheel Polygonization

**Figure 2:**
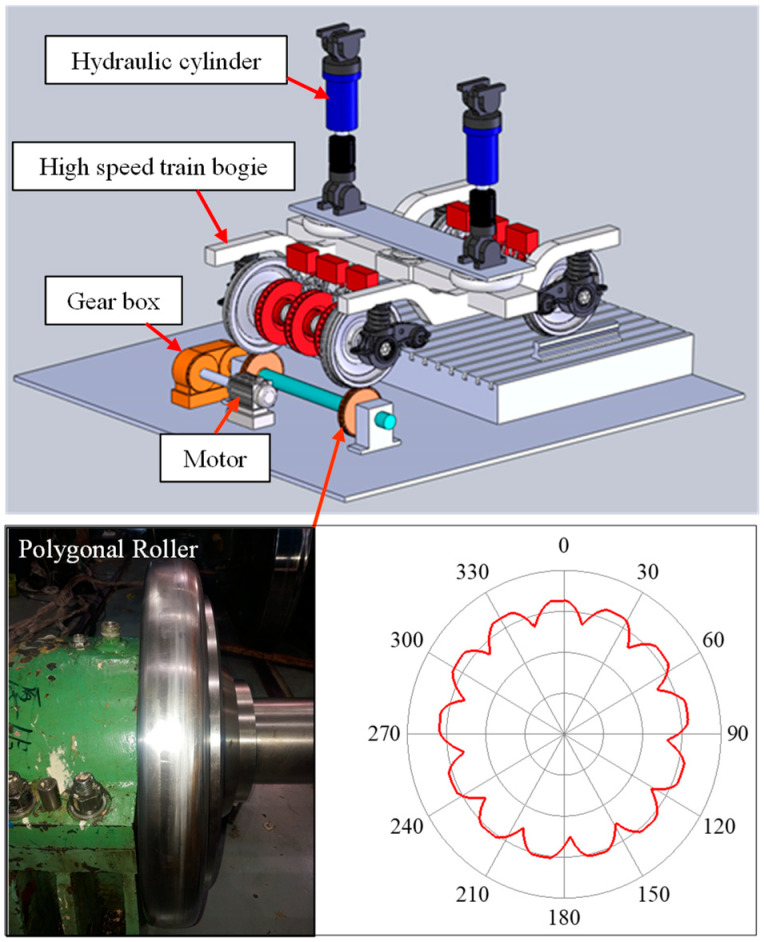
Vibration test bench.

Dynamic characteristics of the front cover were experimentally studied on a test bench, as shown in Figure 2. The roller was out-of-round and a 13th-order polygonal wear was formed along the circumference of the tread, the polygonal roller not only simulated the rail but also transformed the roller polygonization into the wheel polygonization by high-speed rotating under the drive of the motor. The weight of the car body was applied by the hydraulic cylinder; thus, the high-speed advance of the polygonal wheel on the rail was simulated successfully at the test bench [41].

In addition, the polygonal order was proportional to the wheel radius, and radiuses of the roller and wheel were 0.3 m and 0.46 m, respectively; thus, the 13th-order polygonal roller meant the wheel had a 20th-order polygonization. Acceleration measuring points were set at the front cover and axle box body, as shown in Figure 1. Then the sweep frequency test was performed with gradual increase of the roller’s rotational speed, and the maximum linear speed was 320 km/h.

#### 2.1.1. Subharmonic Resonance

**Figure 3:**
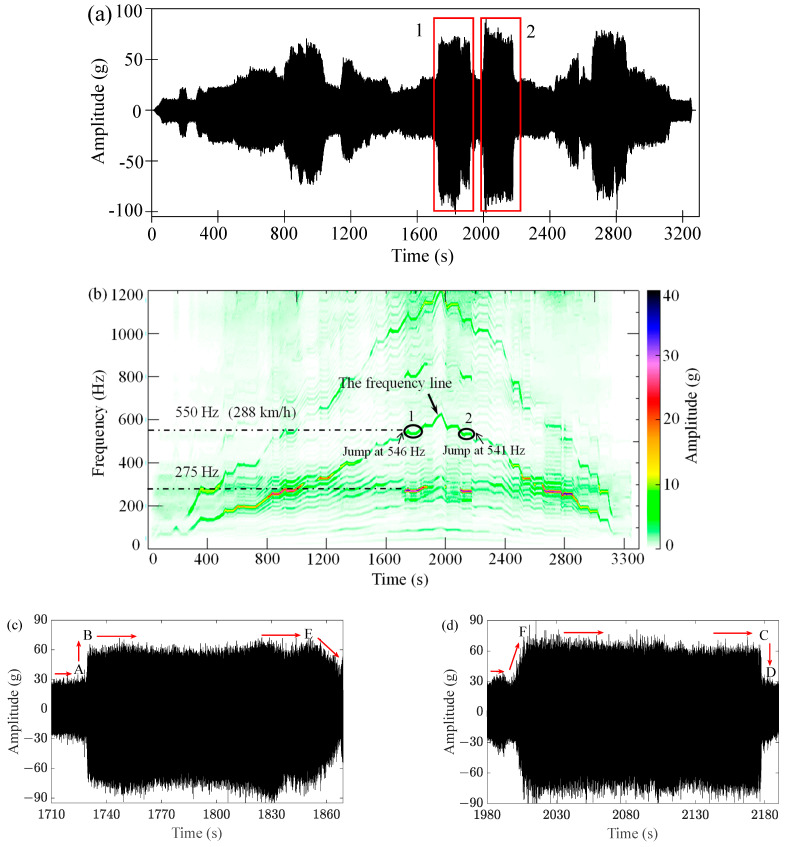
Vertical vibration characteristics of the front cover in bench test. (**a**) Time-domain signal of acceleration; (**b**) time-frequency analysis result; (**c**) jump phenomenon in acceleration process (amplification of the signal in box 1 of Figure 3a); (**d**) jump phenomenon in deceleration process (amplification of the signal in box 2 of Figure 3a).

Figure 3 shows the vertical vibration characteristics of the front cover when the radial deviation of the polygonal roller was 0.075 mm. As shown in Figure 3a, the vibration amplitude was greatly enlarged at region 1 and 2; the corresponding excitation frequency of 20th-order wheel polygonization was also labelled as 1 and 2 in Figure 3b. The relationship between the excitation frequency *f* and linear speed of wheel *v* (m/s) is described in Equation (1):

(1) $$f=\frac{vn}{2\pi r}$$

where *n* = 20 is the polygonal order of wheel; *r* = 0.46 m is the wheel radius. The linear speed of the wheel was 288 km/h at 1 and 2, so the excitation frequency of wheel polygon was 550 Hz according to Equation (1). However, the dominant frequency was around 275 Hz at 1 and 2, which was the natural frequency of the bolted front cover and equal to half the excitation frequency; this vibration characteristic indicated that the front cover was in subharmonic resonance of order 1/2, and 288 km/h was a critical speed.

#### 2.1.2. Superharmonic Resonance

**Figure 4:**
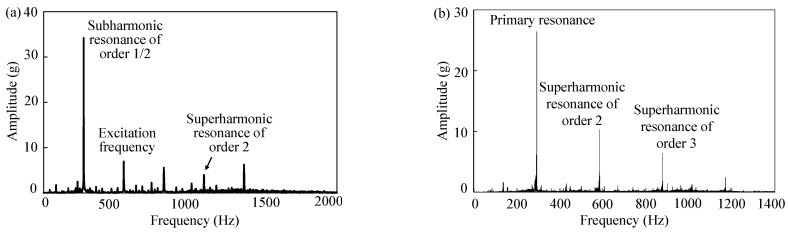
Spectral analysis results of vertical vibration signals of the front cover. Excitation frequency of 20th-order polygonization was (**a**) 550 Hz under wheel radial deviation of 0.075 mm; (**b**) 280 Hz under wheel radial deviation of 0.1 mm.

Spectral analysis results of the signal of Figure 3c are represented in Figure 4a; obviously, superharmonic resonance of order 2 for the front cover also occurred. Figure 4b represents the spectral analysis results of vertical vibration of the front cover under wheel radial deviation of 0.1 mm and excitation frequency of 280 Hz. According to the main frequencies in Figure 4b, the front cover was not only in primary resonance but also in superharmonic resonance of order 2 and 3.

#### 2.1.3. Jump Phenomenon

**Figure 5:**
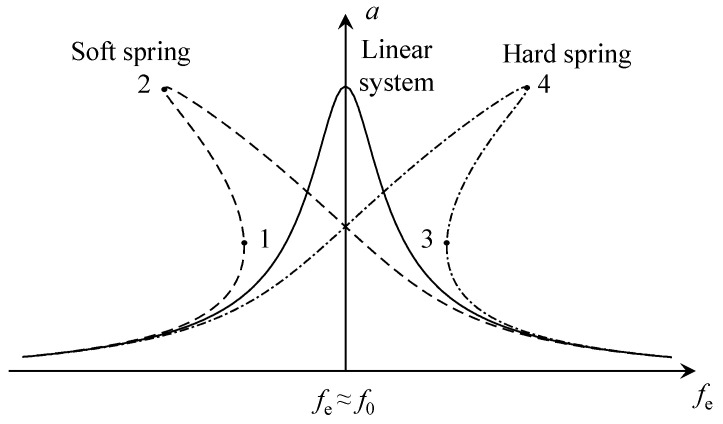
Frequency response curve of linear and nonlinear system.

Other than subharmonic resonance and superharmonic resonance, the jump phenomenon is also a unique characteristic of nonlinear vibration, in which the response of structural vibration has a sudden change under specific external excitation. The essence is due to stiffness softening or stiffness hardening of a system, which are also called soft spring or hard spring. Figure 5 is a simple illustration of resonant response curves of linear and nonlinear systems; *f*_e_, *f*_0_ and *a* refer to excitation frequency, natural frequency and response amplitude respectively. The linear system is resonant when *f*_e_ ≈ *f*_0_, but the soft spring nonlinearity bends the frequency-response curve to the left, while the hard spring nonlinearity bends the curve to the right. Consequently, the vibration amplitude will jump at certain *f*_e_ values (points 1, 2, 3, 4) below and above the natural frequency, respectively.

Figure 3c,d represent the time-domain signals at region 1 and 2 in Figure 3a. Figure 3c shows the jump phenomenon in the acceleration process;. the direction of the arrows represent an increase in speed, the response amplitude jumped from A to B when the wheel speed increased to *v*_1_, which was slightly less than 288 km/h. Speed was constant between B and E, then the wheel speed continued to increase, but the vertical amplitude of the front cover gradually decreased until the maximum test speed of 320 km/h was reached. In contrast, the jump phenomenon in the deceleration process is shown in Figure 3d; while the direction of the arrows represent an decrease in speed, vertical vibration of the front cover gradually increased to maxima at F when the speed was reduced to *v*_2_ (*v*_2_ was slightly smaller than *v*_1_). Speed was constant between F and C, followed by the jump phenomenon from C to D when the speed continued to decrease from *v*_2_. In short, the jump phenomenon of vibration amplitude occurred below the natural frequency, which corresponds to the soft spring curve in Figure 5.

### 2.2. Nonlinear Modeling of the Bolted Front Cover

The front cover was connected by six bolts evenly distributed along the circumference, which can be simplified to a spring-mass-damper system of SDOF. According to Newton’s second law, dynamics of the front cover can be represented by the second-order ordinary differential equation:

(2) $$m\ddot{x}=C(\dot{x},x)\dot{x}+K(\dot{x},x)x+E(t)$$

which is a general model for dynamic behavior analysis and nonlinear parameter identification of the bolted joints [42,43], where *m* = 3 kg is mass of the front cover; the variable $\ddot{x}$ represents acceleration; *E*(*t*) = *F*cos2π*f*_e_*t* is external force from the periodic excitation of wheel polygonization; *f*_e_ is excitation frequency in Hz; $C(\dot{x},x)\dot{x}$ and $K(\dot{x},x)x$ represent the damping force and restoring force, respectively.

According to the analysis results of Section 2.1, vertical vibration of the front cover had the nonlinear characteristics of subharmonic resonances and jump phenomena. It has been proven that subharmonic resonance of order 1/2 and superharmonic resonance of order 2 could be caused by quadratic nonlinear stiffness, while the cubic stiffness nonlinearity took responsibility for occurrence of the superharmonic resonance of order 3 [44], therefore, the restoring force can be expressed as the form of

(3) $$K(\dot{x},x)x=-k_{1}x-k_{2}x^{2}-k_{3}x^{3}$$

The jump phenomenon of a hard spring system can be described by combining the quadratic and cubic nonlinear stiffness as well as linear damping [44]. However, it was found that the jump phenomenon of a soft spring system could occur under the same conditions, which showed that the system with soft spring nonlinearity was more complex than that with hard spring nonlinearity. Therefore, the effect of nonlinear damping was considered in this study, and the damping force had the form of

(4) $$C(\dot{x},x)\dot{x}=c_{1}\dot{x}+c_{2}{\dot{x}}^{2}$$

The governing equation of vertical vibration of the front cover was obtained by substituting Equations (3) and (4) into Equation (2) and eliminating *m*:

(5) $$\ddot{x}+2\mu \dot{x}+4\pi^{2}f_{0}{}^{2}x+\alpha_{2}x^{2}+\alpha_{3}x^{3}+\alpha_{4}{\dot{x}}^{2}=k\cos2\pi f_{e}t$$

where *f*_0_ represents the natural frequency of the derived system in Hz, and the derived system is the corresponding linear system when $\alpha_{2}$, $\alpha_{3}$ and $\alpha_{4}$ are zero; *k* = *F*/*m* is the amplitude of excitation from the axle box body; $\alpha_{2}$ and $\alpha_{3}$ refer to the coefficients of quadratic and cubic stiffness, respectively; μ and $\alpha_{4}$ are coefficients of linear damping and quadratic nonlinear damping, respectively; μ is proportional to the damping ratio *ζ*:

(6) $$\mu =\frac{c_{1}}{2m}=\frac{\zeta \omega }{2m}$$

*ζ* usually ranges from 1% to 10% in engineering, and the damping ratio must be relatively small because of the occurrence of nonlinear vibration; let *ζ* = 1.5%, so μ = 4.3.

The method of multiple scales used to seek a second approximation [45] for the solution of Equation (5) and yields the frequency-response Equation (7) for the non-trivial solution:

(7) $${\left(\varepsilon \Phi_{1}a^{2}-\pi \sigma f_{0}+\varepsilon \Phi_{2}\right)}^{2}-\Phi_{3}^{2}k^{2}+{\left(2\pi f_{0}\mu \right)}^{2}=0$$

(8) $$\Phi_{1}=\frac{3\alpha_{3}}{8}-\frac{5\alpha_{2}^{2}}{48\pi^{2}f_{0}{}^{2}}-\frac{5\alpha_{2}\alpha_{4}}{12}-\frac{\alpha_{4}^{2}}{6},$$

(9) $$\begin{array}{c} \Phi_{2}=\frac{k^{2}}{16\pi^{4}{\left(f_{0}{}^{2}-f_{e}{}^{2}\right)}^{2}}\left[3\alpha_{3}+\frac{\left(\alpha_{2}+4\pi^{2}f_{e}{}^{2}\alpha_{4}+4\pi^{2}f_{0}f_{e}\alpha_{4}\right)\left(\alpha_{2}-4\pi^{2}f_{0}f_{e}\alpha_{4}\right)}{2\pi^{2}f_{e}\left(f_{e}+2f_{0}\right)}\right.\\ -\frac{\alpha_{2}\left(\alpha_{2}+4\pi^{2}f_{e}{}^{2}\alpha_{4}\right)}{2\pi^{2}f_{0}{}^{2}}\left.-\frac{\left(\alpha_{2}+4\pi^{2}f_{0}f_{e}\alpha_{4}\right)\left(\alpha_{2}+12\pi^{2}f_{0}f_{e}\alpha_{4}\right)}{8\pi^{2}f_{0}{}^{2}}\right]-\frac{\mu^{2}}{2} \end{array},$$

(10) $$\begin{array}{ll} \Phi_{3}= & \frac{1}{16\pi^{4}{\left(f_{e}{}^{2}-f_{0}{}^{2}\right)}^{2}}\left\{{\left(\alpha_{2}+4\pi f_{0}f_{e}\alpha_{4}\right)}^{2}\left(1-\frac{\varepsilon^{2}\sigma }{4\pi f_{0}}\right)+\right.\\ \; & \left.{\left(2\pi \varepsilon^{2}\mu f_{e}\right)}^{2}{\left[\frac{\alpha_{2}+4\pi f_{0}f_{e}\alpha_{4}}{2\pi^{2}\left(f_{e}{}^{2}-f_{0}{}^{2}\right)}-\alpha_{4}\right]}^{2}\right\} \end{array}$$

where *a* is the amplitude of frequency response; the parameter ε≪1; *σ* is the detuning parameter, which quantitatively describes the nearness of *f*_e_ to *f*_0_.

**Figure 6:**
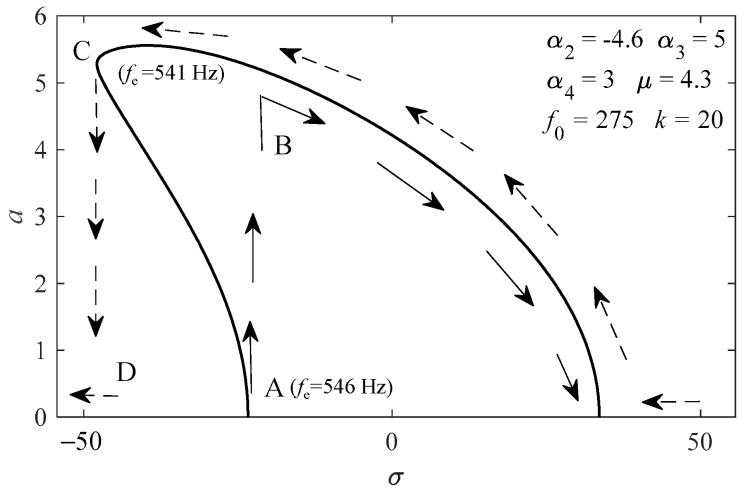
Frequency-response curve of the governing Equation (5).

**Figure 7:**
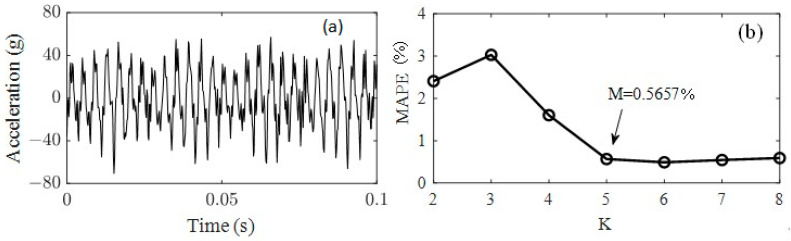
Vertical acceleration signals of the front cover. (**a**) Test signal; (**b**) the optimal number of mode decomposition.

In this study, *ε* = 0.2, *f*_0_ = 275 Hz, and $f_{e}=2f_{0}+\frac{\varepsilon \sigma }{2\pi }$. As shown in Figure 3b, the jump phenomena occurred at *f*_e_ ≈ 545 Hz in the acceleration process and *f*_e_ ≈ 540 Hz in the deceleration process; therefore, the difference of σ was about 25 between the two jump points. It is difficult to determine the coefficient of nonlinear term in both theoretical calculation and experimental analysis; a set of values of $\alpha_{2}$, $\alpha_{3}$ and $\alpha_{4}$ is given to make the frequency response (Figure 6) and acceleration response (Figure 7) as close to reality as possible. As shown in Figure 6, the subharmonic resonance amplitude *a* jumped as the change of *f*_e_. The solid arrows represent the increase process of *f*_e_; *a* jumped upward from A to B when *f*_e_ increased to about 545 Hz, then *a* gradually decreased as *f*_e_ continued to increase. In the decrease process of *f*_e_, which is marked with the dotted arrows, *a* gradually increased as *f*_e_ decreased, and jumped downward from C to D until *f*_e_ decreased to about 540 Hz. It can be seen that the two jump phenomena correspond to the jump phenomena of the acceleration and deceleration process in the bench test (Figure 3c,d), respectively.

### 2.3. Effect of Multiple Parameters on Structural Subharmonic Resonance

In this section, effectiveness of the common prevention method for connecting bolt failure under structural subharmonic resonance was studied by investigating the effect of multiple parameters of Equation (5) on the vertical vibration response of the front cover system.

#### 2.3.1. Parameter Setting

Values of the multiple parameters are shown in Table 1. Curve O is used for comparison, which stands for original values of the parameters that are also the values for Figure 6. In addition, it was found that when the value of $\alpha_{2}$, $\alpha_{3}$ and $\alpha_{4}$ were in the range of (−7.3, −4.3), (−42, 9.9) and (2.76, 4.49), respectively, the jump phenomena would occur.

#### 2.3.2. Comparison between the Test Signal and Numerical Signal

**Figure 8:**
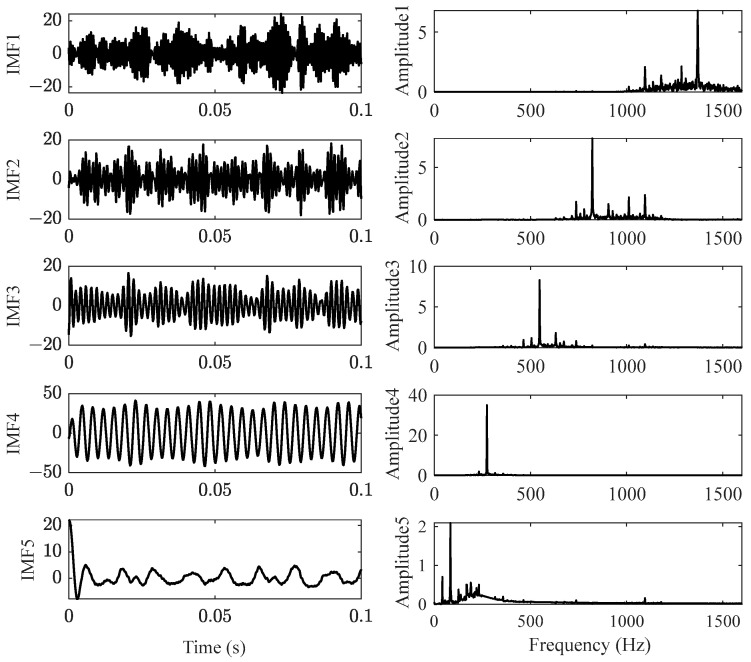
VMD results of the test signal.

Figure 7a is the locally amplified signal of Figure 3c; there are several frequency components for vertical vibration of the front cover. The test acceleration signal was decomposed by VMD to obtain the main frequencies and reduce noise; the VMD function decomposes a signal into a small number *K* (*K* ≥ 2) of narrowband intrinsic mode functions (IMFs) and can effectively avoid modal aliasing and edge effects. To avoid over-decomposition and under-decomposition, the appropriate number *K* can be predicted by the evaluation index of the mean absolute percentage error (MAPE) [46], which represents the ratio of the residual after VMD decomposition to the original signal. In this paper, the minimum *K* value with MAPE value less than 1% was selected as the optimal number of mode decomposition. Through verification, as shown in Figure 7b, it was found that *K* = 5 (MAPE = 0.5657%) was appropriate. As shown in Figure 8, the test signal (Figure 7a) was decomposed to five IMFs; each IMF has a central frequency, and IMF 3 and IMF 4 represent the 550 Hz vibration and 275 Hz vibration, respectively; the former is the excitation frequency of wheel polygonization, while the latter is the subharmonic resonance frequency.

**Figure 9:**
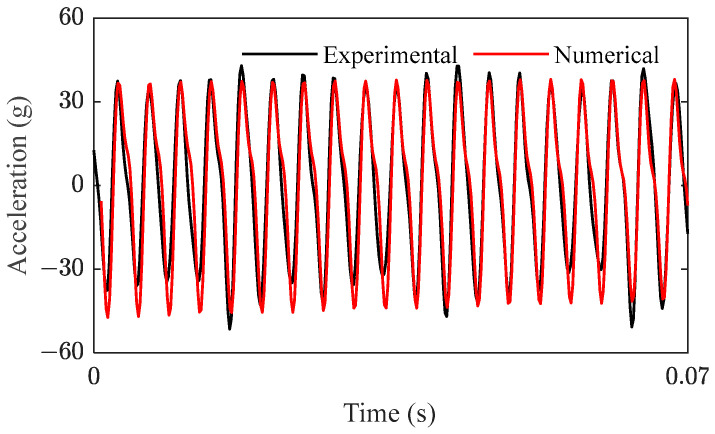
Comparison of the experimental and numerical signal.

IMF 5 can be regarded as a noise signal due to its irregular vibration and small amplitude. For comparison, the test signal of subharmonic resonance of order 1/2 under excitation of 550 Hz was constructed by adding IMF 3 and IMF 4, as shown in Figure 9. The numerical signal was obtained by substituting the parameters of curve O in Table 1 into Equation (5). Both the experimental and numerical vibrations were steady-state motions and have very similar characteristics.

**Figure 10:**
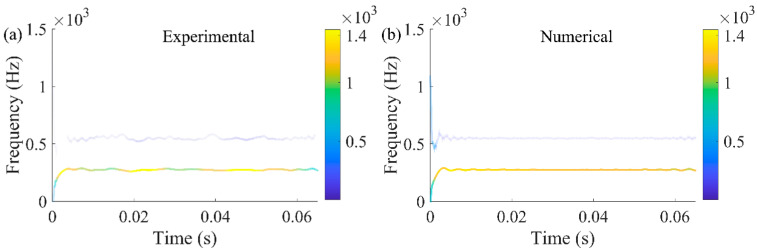
HHT of (**a**) test signal and (**b**) numerical signal.

As shown in Figure 10, the time-frequency spectrums of the two signals were obtained by HHT. The energy distribution showed that both the signals contained frequencies of 275 Hz and 550 Hz, and 275 Hz was the main frequency; in other words, subharmonic resonance of order 1/2 was the most outstanding vibration characteristic, which is also a key point throughout this study. Consequently, it was further proved that the SDOF modeling method is reasonable and feasible to simulate the vertical vibration of the front cover.

#### 2.3.3. Qualitative Analysis for Effect of Multiple Parameters

**Figure 11:**
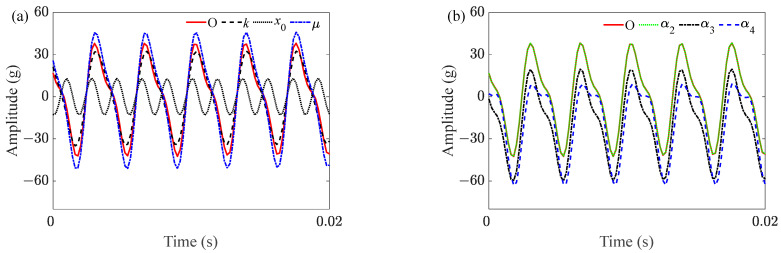
Effect of multiple parameters on vertical resonance of the front cover. (**a**) Effect of excitation amplitude and linear damping; (**b**) effect of nonlinear terms.

In this subsection, the effect of multiple parameters on subharmonic resonance response of the front cover was analyzed. As shown in Figure 11a, it was effective to reduce the vibration amplitude by changing the excitation amplitude and the initial condition. The effect of the linear damping coefficient *μ* on vibration response was closely related to initial conditions; under the nonzero initial condition, the decrease of *μ* increased the subharmonic resonance response. However, subharmonic resonance was not excited in the zero initial condition; even if *μ* was further reduced, the vibration amplitude was much smaller than the response in subharmonic resonance. As shown in Figure 11b, subharmonic resonance was more sensitive to $\alpha_{3}$ and $\alpha_{4}$ than to $\alpha_{2}$. In addition, there is no doubt that *f*_0_ and *f*_e_ have great influence on the subharmonic resonance, but as long as $f_{e}\approx 2f_{0}$, changing the natural frequency of the derived system will only change the cycle period without affecting the occurrence of subharmonic resonance.

#### 2.3.4. Evaluation of the Common Prevention Methods

Essentially, the common prevention methods of adopting spring washers, replacing the lubricating oil with grease, and increasing the preload all increased the friction between interfaces, which is effective for bolt connection under static loads because the friction is more difficult to overcome. However, according to the above analysis results, uncertainty of the effect of damping on a nonlinear system means increasing the preload may not achieve a satisfactory result. Although adequate damping could prevent subharmonic resonance, it is impossible to increase the damping of bolted joints so much by the common prevention methods. In addition, the linear stiffness of the connecting bolt, *k*_1_, can be expressed as

(11) $$k_{1}=\frac{EA}{l}$$

where *E* is Young’s modulus of the bolt material, and *A* and *l* are the sectional area and length of bolt, respectively. When the preload increases, the decrease of *A* and increase of *l* lead to the decrease of bolt connection stiffness *k*_1_. Meanwhile, the natural frequency of the derived system ω, which is proportional to *k*_1_, also decreases. In this case, the change of *f*_0_ caused by preload is so small due to the small variation of *A* and *l* that it is difficult to affect the structural vibration significantly. In other words, the common prevention methods were not effective enough for improving the service life of connection bolts in the front cover because dynamic behavior of the nonlinear system was hard to predict and control. In the following sections, the fatigue life of bolts under structural subharmonic resonance are analyzed and solutions proposed to improve the service life of the connecting bolt.

## 3. Feasibility Analysis for the Stress Simulation Method

Nonlinear characteristics of structure were ignored automatically at the modal-related analysis stage of dynamic stress simulation. Therefore, feasibility of the calculation method was verified in this section. Firstly, axial load measuring bolts were made and calibrated to measure the axial dynamic stress of bolts; moreover, multiaxial acceleration excitations as well as the operational modes of the front cover were also measured from the vibration test. Then, the FE model of the bolted front cover was established, and the mode-based steady-state dynamics analysis was performed to obtain the FRFs (Frequency Response Functions). Finally, the vertical acceleration of the front cover and axial stress of bolts were simulated based on the multiaxial excitations and FRFs.

### 3.1. Theoretical Background of the Stress Simulation Method

In this section, the stress simulation method was studied to calculate accurate structure dynamic stress under resonance and multi-load conditions. Based on the structure dynamic theory and previous research results [22,47], the dynamic stress time history calculation method was adopted in this study, in which the phase information between multi loadings was not ignored. Dynamics of multi-degree-of-freedom systems subjected to time-varying excitation can be governed by the following differential equations:

(12) $$M\ddot{X}(t)+C\dot{X}(t)+KX(t)=f_{e}(t)$$

where *M*, *C*, and *K* are mass, damping and stiffness matrices, respectively; X(t) and $f_{e}(t)$ are displacement and excitation vectors, respectively. Assuming that there are totally *R* modes, the load-stress FRFs can be obtained by Equation (12) [47]:

(13) $$H(\Omega )=E\cdot \sum_{r=1}^{R}\frac{\{\psi_{r}^{\varepsilon }\}{\left\{\varphi_{r}\right\}}^{T}}{k_{r}-\Omega^{2}m_{r}+j\Omega c_{r}}$$

where $m_{r}$, $c_{r}$, $k_{r}$ are the *r*th modal mass, modal damping, and modal stiffness, respectively; Ω is the excitation frequency; $\left\{\varphi_{r}\right\}$ is the real value mode vector; $\left\{\psi_{r}^{\varepsilon }\right\}$ is the *r*th strain mode corresponding to $\left\{\varphi_{r}\right\}$; $j=\sqrt{-1}$; *E* is elastic module.

Due to the front cover being fixed to the axle box body, so accelerations at the end of the axle box body were regarded as the input load. For the acceleration-stress FRF, the phase information needs to be retained, as shown in Equation (15):

(14) $$\begin{array}{ll} H(\Omega ) & =E\cdot \left\{\sum_{r=1}^{R}\frac{\{\psi_{r}^{\varepsilon }\}{\left\{\varphi_{r}\right\}}^{T}}{{\left(k_{r}-\Omega^{2}m_{r}\right)}^{2}+{\left(\Omega c_{r}\right)}^{2}}[(k_{r}-\Omega^{2}m_{r})+j(-\Omega c_{r})]\right\}\\ \; & =E\cdot \left\{\sum_{r=1}^{R}\frac{\left(1-\lambda_{r}^{2}\right)}{k_{er}[{\left(1-\lambda_{r}^{2}\right)}^{2}+{\left(2\zeta_{r}\lambda_{r}\right)}^{2}]}+j\cdot \sum_{r=1}^{R}\frac{-2\zeta_{r}\lambda_{r}}{k_{er}[{\left(1-\lambda_{r}^{2}\right)}^{2}+{\left(2\zeta_{r}\lambda_{r}\right)}^{2}]}\right\}\\ \; & =H^{R}(\Omega )+j\cdot H^{I}(\Omega ) \end{array}$$

where $k_{er}=k_{r}/\{\{\psi_{r}^{\varepsilon }\}{\left\{\varphi_{r}\right\}}^{T}\}$, $\lambda_{r}=\Omega /\omega_{r}$, $\omega_{r}{}^{2}=k_{r}/m_{r}$, $\zeta_{r}=c_{r}/\sqrt{4m_{r}k_{r}}$.

In addition, assuming the length of a discrete acceleration signal *f*(n) is *N*, the FFT of *f*(n) is

(15) $$\begin{array}{ll} F(\Omega ) & =\sum_{n=0}^{N-1}f(n)e^{-j\Omega n}\\ \; & =\sum_{n=0}^{N-1}f(n)\cos n\Omega -j\cdot \sum_{n=0}^{N-1}f(n)\sin n\Omega \\ \; & =F^{R}(\Omega )+j\cdot F^{I}(\Omega ) \end{array}$$

In the case of multi-load input, that is, when the number of external loadings is more than one, and assuming that the number is *k*_e_, all the loadings should be considered to stimulate the stress response; thus, the frequency domain of concerned point stress under the multi-load input condition can be calculated by Equation (16) below:

(16) $$\begin{array}{ll} \sigma (\Omega ) & =\sum_{i=1}^{k_{e}}[F_{i}(\Omega )\cdot H(\Omega )]\\ \; & =\sum_{i=1}^{k_{e}}\left\{[F_{i}{}^{R}(\Omega )H^{R}(\Omega )-F_{i}{}^{I}(\Omega )H^{I}(\Omega )]+j\cdot [F_{i}{}^{R}(\Omega )H^{I}(\Omega )-F_{i}{}^{I}(\Omega )H^{R}(\Omega )]\right\} \end{array}$$

Then, the time history stress at the concerned point can be calculated by IFFT:

(17) $$\sigma (N)=\frac{1}{N}\sum_{n=0}^{N-1}\left[\sigma (\Omega )e^{j\Omega n}\right]$$

σ(N) is the simulated dynamic stress time history, which is used for fatigue life prediction.

### 3.2. Acquisition of Vibration Signals

#### 3.2.1. Fabrication and Calibration of Axial Load Measuring Bolt

A nonlinear connection such as a bolted joint is usually simplified as a linear system because the accurate nonlinear model is difficult to be established, but the premise is that the accuracy of calculation results can be guaranteed. In this study, damping of the bolted joint was assumed to be linear; therefore, feasibility of the linear stress simulation method was assessed in terms of the axial and transverse stiffness of the bolted joint.

**Figure 12:**
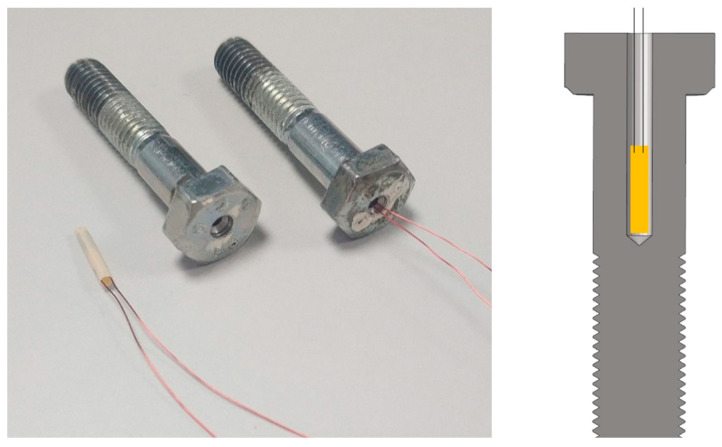
Axial stress measuring bolt.

The axial stiffness could be assessed by certifying the bolt axial stress. In order to measure the axial bolt stress in the vibration test, axial stress measuring bolts were made, as shown in Figure 12. Firstly, a hole with diameter of 2.2 mm and an appropriate depth was drilled in the center of the bolt. Then, an LB11 strain gauge was attached to inside of the bolt by a hot-curing epoxy resin adhesive. LB11 is a cylindrical strain gauge to measure strain, force, and vibration in screws or bolts [48].

Drilling reduced the effective cross section and durability of the bolt, which resulted in that the stress-strain relationship could not be depicted with the classical material mechanics formula. Hence, calibration for the drilled bolts was conducted by a strain gauge load cell for tension and compression. Calibration for each bolt was repeated three times, and the results had great consistency. The relationship between the micro strain *ε_m_* (μm/m) and the bolt axial stress *S_a_* (MPa) is shown in Equation (18).

(18) $$S_{a}=\frac{7.9704\varepsilon_{m}-2.813}{A_{e}}$$

where *A*_e_ is the effective sectional area of the bolt shank, which should minus the area of the drilled hole.

#### 3.2.2. Measurement of Multiaxial Excitations and Bolt Axial Load Variation

As shown in Figure 1, triaxial accelerometers were set at the end of the front cover and the axle box body where near the front cover, two axial load measuring bolts were assembled symmetrically at the front cover and tightened with the torque of 20 Nm. Only vibrations of the axle box body were considered as multiaxial external loads because almost all the external excitations of the front cover came from the axle box body; the multiload can be divided into the longitudinal acceleration, the lateral acceleration, and the vertical acceleration.

**Figure 13:**
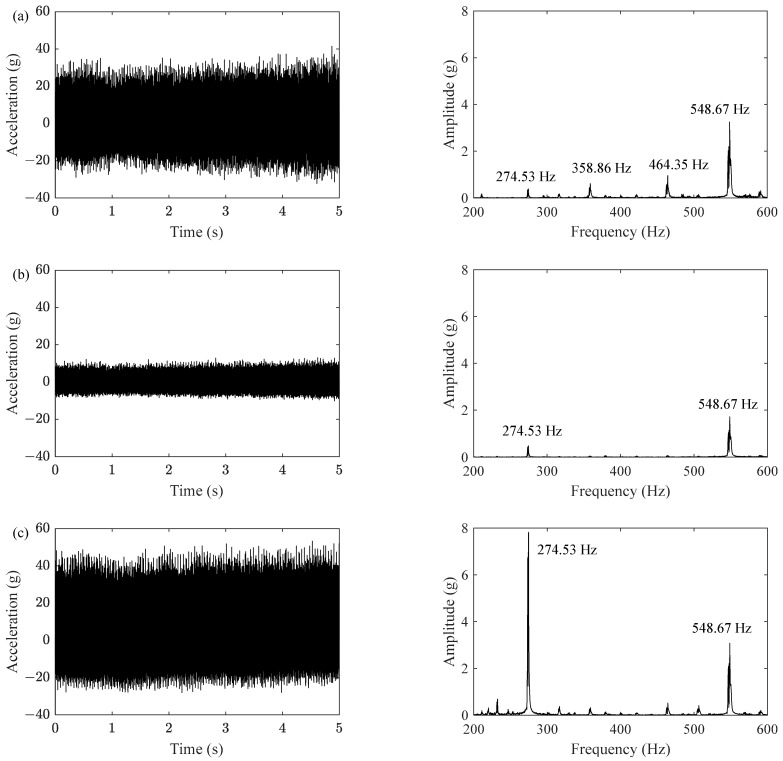
Multiaxial acceleration excitations in time domain and frequency domain. (**a**) Longitudinal excitation; (**b**) lateral excitation; (**c**) vertical excitation.

The multiaxial excitations of the front cover are shown in Figure 13: the left is the time domain signal at the running speed of 288 km/h, while the right is the FFT result of the left. The largest amplitude of vertical vibration was 40 g, followed by longitudinal vibration, and the smallest was lateral vibration. The dominant frequencies of the three accelerations were all multiples of 27.49 Hz, which is the theoretical rotational frequency of the wheel that is equal to wheel perimeter divided by linear speed of 288 km/h and is also the frequency when *n* = 1 in Equation (1).

**Figure 14:**
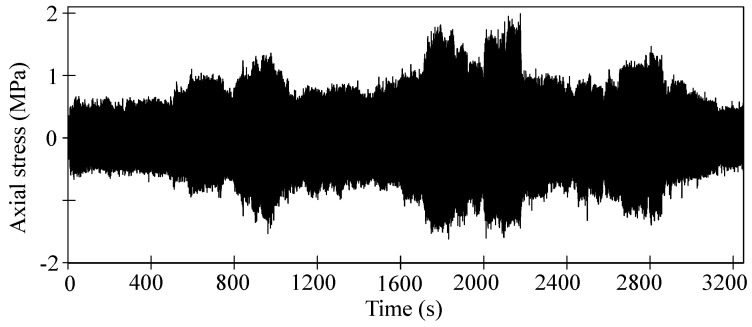
Time history of the bolt axial stress.

As shown in Figure 14, the dynamic axial stress of the bolt was obtained in accordance with the micro-strain measured by LB11 strain gauge and Equation (18). Obviously, the amplitude of dynamic axial stress also increases in the subharmonic resonance state and changes between ±2 MPa. It is noticeable that the micro-strain measurement started after bolt tightening, so axial stress caused by preloading is not included in Figure 14.

#### 3.2.3. Modal Test of the Front Cover

In this section, the vibration mode of the front cover was obtained by modal test and used to verify the correctness of the subsequent numerical modeling. OMA (Operational Modal Analysis) rather than EMA (Experimental Modal Analysis) was chosen due to the practical advantages [41,49,50,51], which is preferable for in-operation structures because the output-only data is needed and the test structure remains in its normal in-operation condition during the test. More importantly, OMA is quite suitable for structures with nonlinear characteristics.

**Figure 15:**
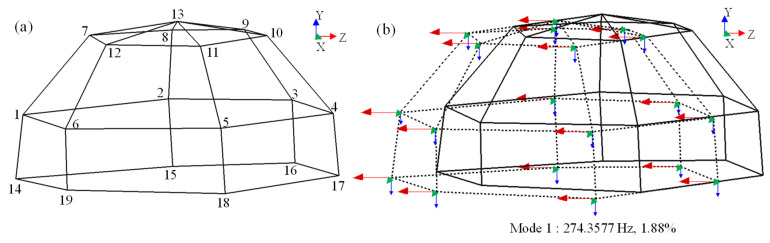
OMA results of the bolted front cover. (**a**) The analytical model composed of the acceleration measuring points; (**b**) the first-order mode.

As shown in Figure 15a, 19 measuring points of acceleration were set on the external surface of the front cover (1–13) and close to the connection bolts (14–19). Then, the frequency sweep test was carried out as in Section 2.1 to obtain the vibration signals in three directions. Finally, modal analysis was carried out in the vibration control system of Simcenter Testlab according to the acceleration signals. As shown in Figure 15b, the solid line model is the undeformed model, while the dotted line model represents the deformed model, and the first-order modal frequency and modal damping were 274.36 Hz and 1.88%, respectively. There are three arrows with different colors at each measuring point; the green, blue, and red arrows represent the vibrations in the x, y, and z directions, respectively. The size of the arrow represents the vibration intensity; obviously, the front cover mainly vibrated in the z direction, that is, perpendicular to the axis of connection bolt, which means that the bolts were mainly subjected to transverse loads.

### 3.3. Finite Element Analysis

#### 3.3.1. Finite Element Modeling

**Figure 16:**
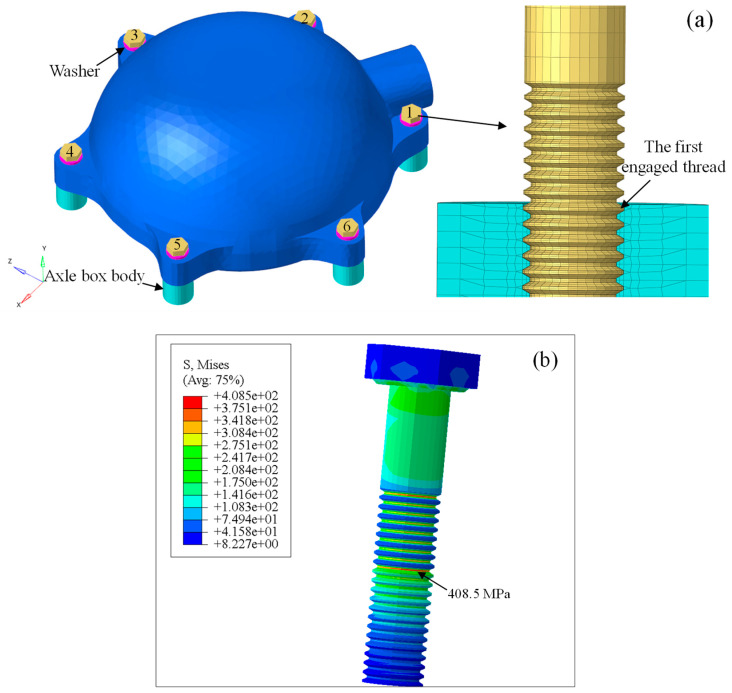
(**a**) FE model of the front cover; (**b**) stress distribution of the preloaded bolt.

As shown in Figure 16a, the three-dimensional FE model of the front cover was established in the CAE software of HyperMesh 2019. The numbers 1-6 refer to the six connecting bolts, the external threads of the bolts and internal threads of the axle box body were constructed with the thread pitch of 1.25 mm. The front cover was divided by tetrahedron elements with type of solid 185, while the bolts, washers and cylinders were divided by the mixture of pentahedrons and hexahedrons. Since the axle box body was assumed to be rigid, only a small region around the tapped hole was modeled, and the external nodes were constrained. In addition, contact elements were overlaid on contact regions between the bolt head and washer, the washer and front cover, the front cover and axle box body, as well as between the internal and external threads. The connecting bolts were unlubricated galvanized bolts, which fit into to the friction coefficient class C of Table A5 in VDI 2230 [52], so the friction coefficients range from 0.14 to 0.24. In this study, the friction coefficients were set to 0.18.

Preload of 11,000 N was applied at shank for each bolt in ABAQUS 2021, followed by a general static analysis; convergence of the contact was calculated by the Newton-Raphson method. Stress distribution on the bolt after preloading is shown in Figure 16b; the maximum stress was 408.5 MPa, which is lower than the material yield limit (640 MPa) of grade 8.8 bolt. Moreover, the maximum stress occurred at the root of the first engaged thread, which is reasonable due to the high notch effect at the first load-bearing thread turn of the bolt [52]. The preload calculation results show that FE modeling of the front cover was reasonable.

#### 3.3.2. Modal Analysis

Modal analysis of the bolted front cover was carried out after the preload calculation; the modes were extracted by the block Lanczos method. The first four order modal frequencies are listed in Table 2, and the first two were around 275 Hz, which further proves that subharmonic resonance of order 1/2 did occur when the excitation frequency was 550 Hz.

**Figure 17:**
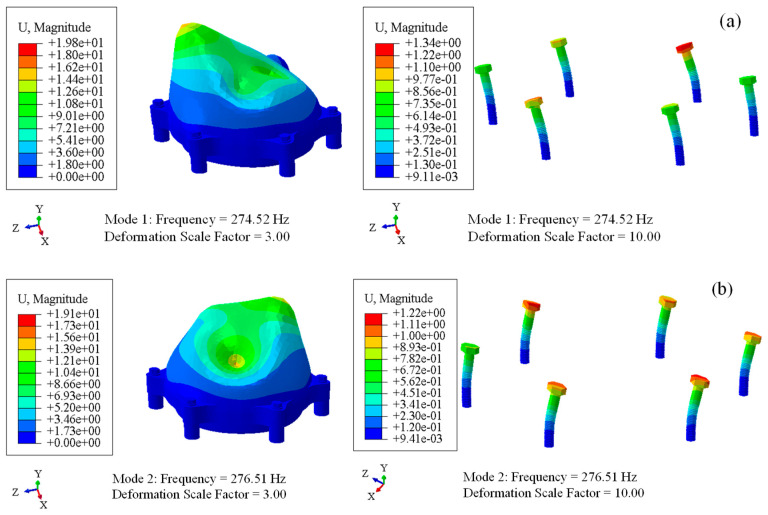
Modal analysis results of the front cover and bolts. (**a**) the first-order mode; (**b**) the second-order mode.

As shown in Figure 17, the first two modes of the connection bolts were the first-order bending modes; in other words, the front cover vibrated in the z direction and the bolts were subjected to transverse loads. The mode frequencies and mode shapes of the first two modes were in good agreement with the OMA results, so validity of the FE model was further proved.

#### 3.3.3. FRF Calculation

Mode-based steady-state dynamics analysis was conducted to calculate the FRFs, which included the real and imaginary parts. A white noise load was regarded as the base excitation input, the frequency band of white noise was from 200 Hz to 600 Hz, which contains the frequencies of interest in this study. In addition to the accurate modal frequency and modal shape, accurate modal damping was also needed for accurate calculation of FRFs. Therefore, the damping ratio 1.88% of OMA was adopted for the first two calculation modes.

**Figure 18:**
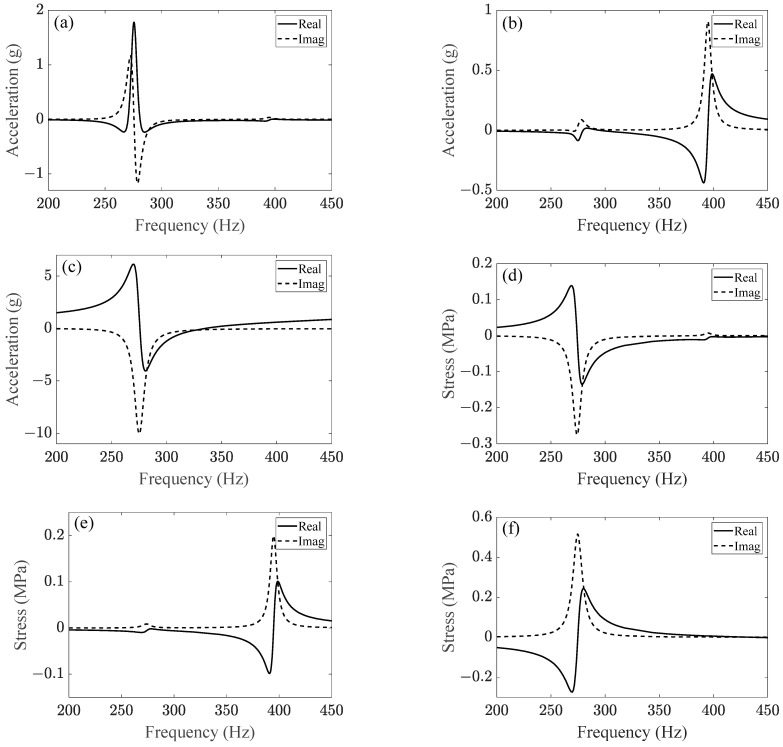
(**a**–**c**) represent vertical acceleration FRFs of the front cover under the excitation of longitudinal load, lateral load, and vertical load, respectively; (**d**–**f**) represent axial stress FRFs at bolt shank under the excitation of longitudinal load, lateral load, and vertical load, respectively.

Feasibility of the linear stress simulation method in axial direction of the bolted joint can be verified easily because bolt axial stress can be measured directly, but transverse stress of bolt was difficult to be measured; therefore, the method was verified in the transverse direction of the bolted joint by verifying the vertical vibration of the front cover. The vertical acceleration FRFs of the front cover are shown in Figure 18a–c; the main frequency was about 275 Hz under longitudinal and vertical excitation, which is close to the first-order bending modal frequency of bolts, while the main frequency was about 395 Hz under the lateral excitation. Simultaneously, the axial stress FRFs of the bolt shank were also calculated to simulate the axle stress of bolts, as shown in Figure 18d–f.

### 3.4. Acceleration and Stress Simulation of the Bolted Front Cover

**Figure 19:**
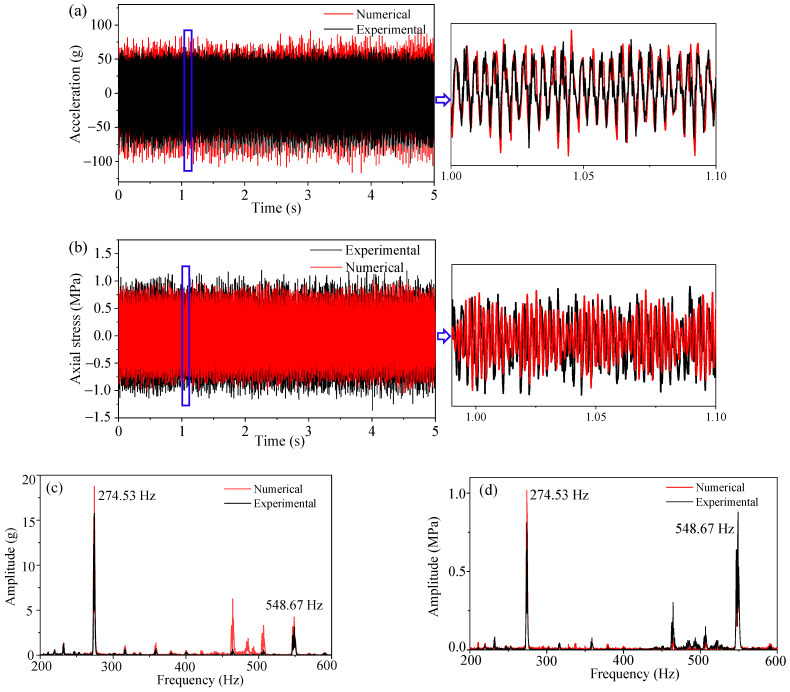
Comparison between the experimental and numerical results. (**a**,**b**) are time domain signals of the front cover and bolt, respectively; (**c**,**d**) are frequency domain signals of the front cover and bolt, respectively.

In this section, dynamic data in the frequency domain are calculated by the frequency domain signals of the multiaxial excitations in Figure 13 and FRFs in Figure 18. Then, dynamic time history can be acquired by Inverse FFT (IFFT) of the frequency domain data. A comparison between the simulation results and test results is shown in Figure 19. As shown in Figure 19a,b, the numerical signals were consistent with the experimental signals. Figure 19c,d show that both the experimental and numerical signals had the dominant frequencies of 274.53 Hz and 548.67 Hz, which corresponded to the first-order bending modal frequency of bolts and the excitation frequency of the 20th-order wheel polygon, respectively.

In summary, the consistency between experimental and numerical results for vertical acceleration and axial stress demonstrates that the linear stress simulation method is able to ensure the calculation accuracy. In other words, this method is feasible for dynamic stress simulation under nonlinear vibration states.

## 4. Analysis of Multiaxial Fatigue Behavior of Connecting Bolt

After feasibility of the stress simulation method was proved, multiaxial fatigue behavior was analyzed in this section. Transverse and axial resonance stresses at the root of the first engaged thread of connecting bolts were calculated firstly. Then, the multiaxial fatigue analysis criterion was determined. Finally, load change cycles were calculated with the rain-flow counting method, and the bolt fatigue life was predicted.

### 4.1. Calculation of Dynamic Stress at Bolt Thread

**Figure 20:**
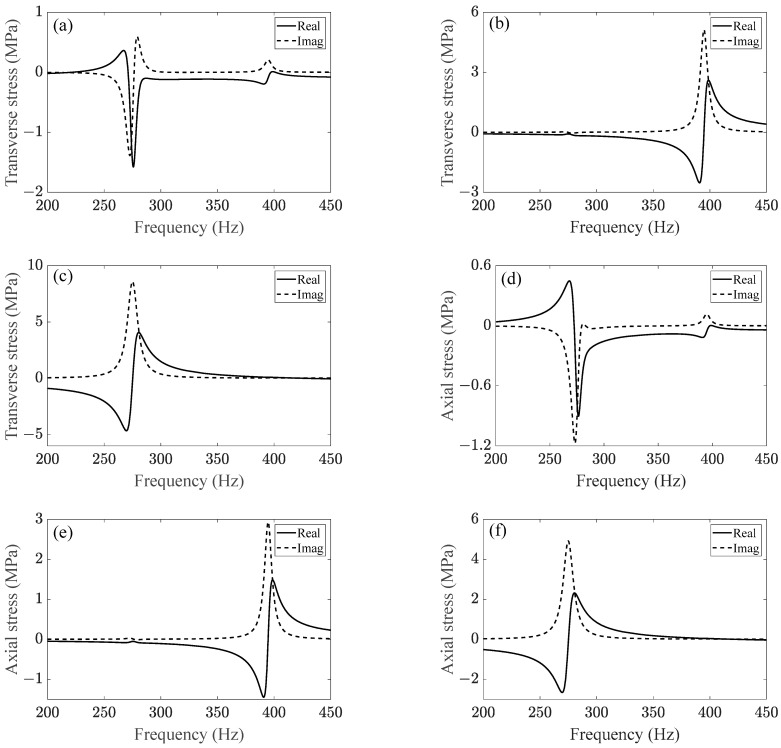
(**a**–**c**) represent transverse stress FRFs at thread root under the excitation of longitudinal load, lateral load, and vertical load, respectively; (**d**–**f**) represent axial stress FRFs at thread root under the excitation of longitudinal load, lateral load, and vertical load, respectively.

As it is the most dangerous location, resonance stress at the first engaged thread must be evaluated. Figure 20a–c shows the transverse stress FRFs of node 233,773 at the first engaged thread of bolt 2 (Figure 16a). Obviously, the 275 Hz vibration was most sensitive to the vertical excitation. The lateral excitation easily excited the 395 Hz vibration but had little effect on the 275 Hz vibration. In other words, subharmonic resonance of order 1/2 of the front cover was mainly aroused by the vertical vibration.

Figure 20d–f show the axial stress FRFs of node 233,773. It is obvious that the axial stress response at node 233,773 was much larger than the response at the bolt shank (Figure 18d–f). Similarly, 275 Hz vibration was more sensitive to vertical and longitudinal excitations, while 395 Hz vibration was more sensitive to lateral excitation. It means that the axial deformation at node 233,773 under subharmonic resonance was not originated from the axial excitation but from the transverse excitations.

**Figure 21:**
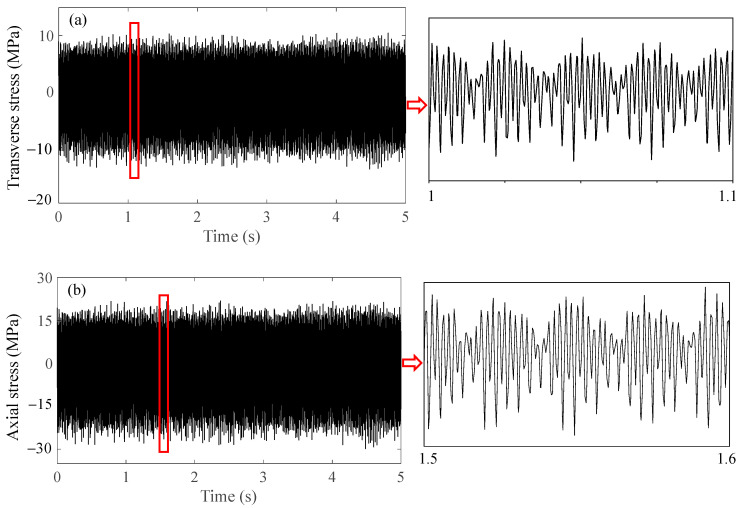
**Dynamic stress simulation results and local amplification.** (**a**) transverse dynamic stress and (**b**) axial dynamic stress at thread root.

Simulation of the transverse dynamic stress of node 233,773 is shown in Figure 21a. The maximum stress range of the transverse stress was around 20 MPa, which was much larger than the axial stress range at the bolt shank (Figure 19b). As shown in Figure 21b, the tensile stress at the root of the bolt thread could not be ignored due to the bending mode; the maximum range of the axial dynamic stress of node 233,773 was around 45 MPa, which was much larger than the stress range at the bolt shank as well. It is obvious that the axial stress at the bolt thread was more predominant than the transverse stress, which would accelerate fatigue-crack propagation [24,25].

### 4.2. Analysis of Bolt Fatigue Strength

#### 4.2.1. Criteria of Stresses Combination and Fatigue Life Calculation

There are some stress combination techniques for multiaxial stresses, such as the critical plane approach and the octahedral shear stress criterion; the latter is also often called either the von Mises or the distortion energy criterion. The octahedral stress criterion is work for in-phase or proportional loading, which is suitable for bolt fatigue analysis in this study because the stresses were all caused by the excitation of 20th-order wheel polygonization. There are three main processes to evaluate fatigue life by the octahedral stress criterion: (1) cycle counting for the stress load history, (2) calculation of equivalent stress for multiaxial stresses, and (3) prediction of fatigue life. The fatigue analysis criterion was firstly represented in this subsection.

According to the octahedral stress criterion, the equivalent stress can be expressed as

(19) $$\sigma^{e}=\frac{1}{\sqrt{2}}\sqrt{{\left(\sigma_{1}-\sigma_{2}\right)}^{2}+{\left(\sigma_{2}-\sigma_{3}\right)}^{2}+{\left(\sigma_{3}-\sigma_{1}\right)}^{2}}$$

where *σ*_1_, *σ*_2_, and *σ*_3_ are principal stresses. The amplitudes of the principal stresses, *σ*_1a_, *σ*_2a_, and *σ*_3a_, can then be employed to compute an equivalent stress amplitude $\sigma_{a}{}^{e}$ by using the similar relationship as Equation (19). In addition, consider the effect of mean stresses; an equivalent mean stress $\sigma_{m}{}^{e}$ can be calculated from the mean stresses in the three principal directions. Therefore, the cyclic stressing can be specified as

(20) $$\left\{\begin{array}{l} \sigma_{a}{}^{e}=\frac{1}{\sqrt{2}}\sqrt{{\left(\sigma_{1a}-\sigma_{2a}\right)}^{2}+{\left(\sigma_{2a}-\sigma_{3a}\right)}^{2}+{\left(\sigma_{3a}-\sigma_{1a}\right)}^{2}}\\ \sigma_{m}{}^{e}=\sigma_{1m}+\sigma_{2m}+\sigma_{3m} \end{array}\right.$$

Equation (20) can be analogized to obtain the equivalent stress amplitude and equivalent mean stress for any convenient coordinate axes:

(21) $$\left\{\begin{array}{l} \sigma_{a}{}^{e}=\frac{1}{\sqrt{2}}\sqrt{{\left(\sigma_{xa}-\sigma_{ya}\right)}^{2}+{\left(\sigma_{ya}-\sigma_{za}\right)}^{2}+{\left(\sigma_{za}-\sigma_{xa}\right)}^{2}+6(\tau_{xya}^{2}+\tau_{yza}^{2}+\tau_{zxa}^{2})}\\ \sigma_{m}{}^{e}=\sigma_{xm}+\sigma_{ym}+\sigma_{zm} \end{array}\right.$$

According to the previous analysis, $\sigma_{y}$ and $\sigma_{z}$ at the bolt thread root were obtained in Section 4.1 (Figure 21); $\sigma_{x}$ was neglected for vertical vibration of the front cover, and the shear stresses were also neglected. Thus, equivalent amplitude and mean value of bolt stresses can be expressed as

(22) $$\left\{\begin{array}{l} \sigma_{a}{}^{e}=\frac{1}{\sqrt{2}}\sqrt{\sigma_{ya}{}^{2}+\sigma_{za}{}^{2}+{\left(\sigma_{ya}-\sigma_{za}\right)}^{2}}\\ \sigma_{m}{}^{e}=\sigma_{ym}+\sigma_{zm} \end{array}\right.$$

An equivalent stress amplitude of the completely reversed uniaxial stress can be obtained by combining $\sigma_{a}{}^{e}$ and $\sigma_{m}{}^{e}$:

(23) $$\sigma_{ar}{}^{e}=\frac{\sigma_{a}{}^{e}}{1-\frac{\sigma_{m}{}^{e}}{\sigma_{f}{}^{\prime }}}$$

where $\sigma_{f}{}^{\prime }$ is a constant and approximately equal to the true fracture strength of material. In this study, $\sigma_{f}{}^{\prime }$= 1200 MPa.

The technical committee of the BS standard has determined several S-N curves suitable for fatigue life calculation of bolted joints through fatigue tests under various load conditions and published them in EN1993-1-9:2005 [53]. According to the GL2010 standard [54], for bolts rolled before heat treatment and subjected to tension and bending loads, the S-N curve for category 71 in EN1993-1-9:2005 is applicable. For constant amplitude stress ranges, the S-N curve has the form as follows:

(24) $${\left(\Delta \sigma_{a}\right)}^{m}N_{f}={\left(\Delta \sigma_{c}\right)}^{m}\times 2\times 10^{6}$$

where $\Delta \sigma_{a}$ is cycling stress range, $N_{f}$ is the number of cycles to fatigue failure, *m* = 3, and the number $\Delta \sigma_{c}$ = 71 represents the reference value of fatigue strength for the curve of category 71 at 2 million cycles. The S-N curve expressed in stress amplitude (half the stress range) is shown below:

(25) $${\left(\sigma_{ar}{}^{e}\right)}^{3}N_{f}=8.95\times 10^{10}$$

Finally, the fatigue life can be predicted according to Miner’s cumulative damage theory.

#### 4.2.2. Fatigue Life Prediction of Bolt

**Figure 22:**
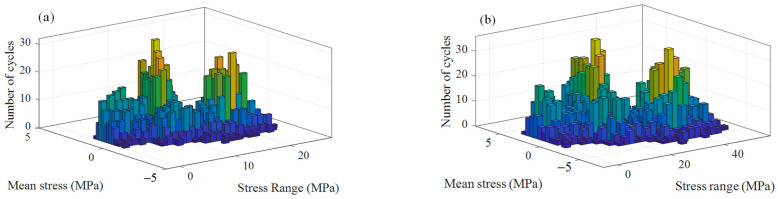
Rain-flow count results for (**a**) transverse stress time history and (**b**) axial stress time history of node 233773.

The number of cycles necessary to break the structure depends on the cycle amplitude and mean value of stress. The rain-flow cycle counting method is an effective method for variable amplitude multiaxial loading which can identify all the stress cycles. According to the rain-flow count, there were totally 2743 different stress cycles and half-cycles for both the transverse stress and axial stress; the number of cycles for each cycle stress was *N*_i_ = 1 or 1/2. The stress ranges and mean stresses are shown in Figure 22.

An equivalent stress amplitude $\sigma_{a}{}^{e}$ and equivalent mean stress $\sigma_{m}{}^{e}$ were obtained by the 2743 different stress ranges and mean stresses according to Equation (22). Then, the equivalent stress amplitude $\sigma_{ar}{}^{e}$ for fatigue calculation was obtained by Equation (23). According to Equation (25), the failure cycle $N_{fi}$ that corresponded to each *N*_i_ was calculated. After getting all the 2743 failure cycles, the accumulation at the root of the first engaged bolt thread caused by transverse and axial cyclic stress can be obtained by Equation (26).

(26) $$D=\sum_{i=1}^{2743}\frac{N_{i}}{N_{fi}}$$

It was calculated that $D=5.18\times 10^{-6}$, which represents the damage at the thread root caused by the multiaxial excitation signal of 5 s length, so fatigue life of the bolt was $t=5/(5.18\times 10^{-6}\times 3600)=26.8$ h, which means that fatigue failure of the bolt would occur if only the subharmonic resonance state of the front cover lasted for more than 26.8 h.

## 5. Method for Improving Fatigue Life of Connecting Bolt

According to the above fatigue analysis results, bolts at the axle box front cover easily failed around the linear speed of 288 km/h after the stable 20th-order polygonal wheel formed. Reprofiling is the most effective way to eliminate the wheel polygonization-induced resonance at present, and 250,000 km is a fixed reprofiling interval for Chinese high-speed trains. Wang et al. [55] reported that vertical vibration of the axle box increased slowly in the early 150,000 km and rapidly in the late 100,000 km, which indicated rapid growth of the polygonization amplitude. Moreover, 280 km/h to 300 km/h is a common running speed range for high-speed trains in China. Therefore, there is a high probability of fatigue failure for connecting bolts of the front cover during a reprofiling interval.

**Figure 23:**
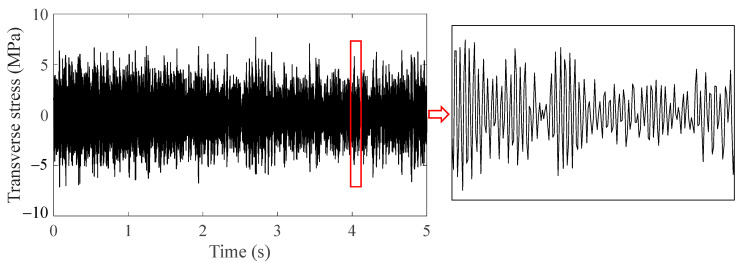
Transverse stress at the thread root and local amplification when there was no wheel polygonization.

Subharmonic resonance of the front cover must be avoided to prevent the multiaxial vibration fatigue of bolts. There are two methods to improve fatigue life of bolts. One is to optimize the structure of the front cover so that the first-order modal frequency is greater than 290 Hz, which is half the excitation frequency of the 20th-order wheel polygon at the running speed of 300 km/h. Nevertheless, modifying the structure of a widely used component is costly. The other method is to prevent the formation of 20th-order wheel polygonization. In order to explore the fatigue behavior of bolts with no wheel polygonization, a sweep frequency test under the condition of wheel tread defect of wheel-flat but no polygonization was carried out. The reason why the wheel with defects was used instead of the new wheel after reprofiling was that the wheel would not be in a perfect state in most of the running time, so a working condition that was inferior to the new reprofiled wheel but better than the polygonal wheel was used. The same frequency sweep test was carried out as previously. The maximum vibrational amplitude of the multiaxial acceleration loadings from the axle box body was around 20 g at the linear speed range of 280–300 km/h, and the front cover was not in resonance; thus, the stress caused by bending modes of the bolt was ignored under the circumstances, and only the effect of transverse stress on bolt fatigue life was considered. The results of transverse stress simulation at the first engaged thread root and local amplification are shown in Figure 23. Obviously, there was an aperiodic vibration with smaller transverse stress amplitude in comparison with the resonance stress in Figure 21a.

The fatigue life of bolts was calculated by the same method as in Section 4. Accumulation caused by transverse vibration at the root of the first engaged thread was $D_{\tau }=1.95\times 10^{-9}$, so the fatigue life of the bolt was more than 700 thousand hours, which means the probability of bolt fatigue failure decreases rapidly as long as the front cover is not in resonance, even though the wheel tread has defects. Hence, shortening the reprofiling interval to reduce the polygonal wear could effectively improve the fatigue life of bolts.

## 6. Conclusions

A linear method based on frequency response analysis was first used to calculate the bolt resonance stress in this work. It was proved that the method was feasible and accurate enough for dynamic stress simulation, which provides a practical way for multiaxial fatigue behavior analysis of bolts under complicated running conditions of high-speed trains. Several conclusions were drawn as follows:

1. The SODF nonlinear modeling method is reasonable for the bolted front cover. The common prevention methods for bolt failure were theoretically proved ineffective under structural subharmonic resonance of order 1/2.
2. The results of bolt stress measurement show that the dynamic behaviors of bolt under a nonlinear vibration state should be assessed due to the increasing of bolt stress.
3. Although the vibration amplitude of the front cover in the direction of the bolt axis was small, the axial resonance stress at the bolt thread’s root could not be neglected due to the first-order bending modes of bolts.
4. Feasibility of the linear stress simulation method was proved in terms of the transverse stiffness and axial stiffness of the bolted joint. It indicated that the method was accurate enough to simulate the subharmonic resonance stress of bolts even if the nonlinearity of the bolted joint was ignored.
5. Resonance stresses at the root of the first engaged bolt thread were much larger than the resonance stress at the bolt shank, and the axial resonance stress was more predominant at the bolt thread than the transverse resonance stress.
6. Since the multiaxial stresses were caused by the homologous excitations, the octahedral shear stress criterion was suitable for equivalent stress calculation. The fatigue life of the bolt was about 26.8 h, which means that the connecting bolts were prone to multiaxial fatigue failure when the front cover was in subharmonic resonance of order 1/2 for a long time.
7. The fatigue life of the bolts is greatly improved when the front cover is not in subharmonic resonance. Consequently, the probability of fatigue failure of bolts could be reduced effectively by shortening the reprofiling interval to reduce the wear of the polygon.

The fatigue life analysis method of bolts adopted in this study has great practical significance for safety monitoring in engineering; it is easy to evaluate the reliability of the bolted joint under various vibration conditions. However, this simulation method neglects the effects of nonlinear factors and is not suitable for strongly nonlinear systems; therefore, a more universal stress simulation method should be explored in subsequent studies. In addition, the octahedral shear stress criterion is not suitable for non-proportional loading, and further study should be advanced for a more universal method, such as the critical plane approach.

## Figures and Tables

**Table 1:** Parameter table.

Curve Number	Linear Damping Coefficient *μ*	Excitation Amplitude *k* (g)	Nonlinear Coefficients			Initial Condition *x*_0_
			*α*_2_	*α*_3_	*α*_4_	
O	4.3	20	−4.6	5	3	(0.03 0.1)
*μ*	3.5	20	−4.6	5	3	(0.03 0.1)
*k*	4.3	12	−4.6	5	3	(0.03 0.1)
*α*_2_	4.3	20	−7	5	3	(0.03 0.1)
*α*_3_	4.3	20	−4.6	8	3	(0.03 0.1)
*α*_4_	4.3	20	−4.6	5	4.4	(0.03 0.1)
*x*_0_	2	20	−4.6	5	3	(0 0)

**Table 2:** Modal analysis results.

Mode	1	2	3	4
Frequency (Hz)	274.52	276.51	394.80	614.65

## Data Availability

The data are not publicly available due to confidentiality agreements of the Institutional.

## Nomenclature

EMA	Experimental modal analysis	$H^{I}(\Omega )$	the imaginary part of *H*(*Ω*)
EMD	empirical mode decomposition	HHT	Hilbert-Huang transform
*f*(n)	the discrete acceleration excitation signal	IMF	intrinsic mode functions
*F*(*Ω*)	FFT result of *f*(n)	*k*_e_	number of external loadings
$F^{R}(\Omega )$	the real part of *F*(*Ω*)	MAPE	mean absolute percentage error
$F^{I}(\Omega )$	the imaginary part of *F*(*Ω*)	*N*	length of *f*(n)
FE	finite element	OMA	operational modal analysis
FEA	finite element analysis	SDOF	single degree of freedom
FEM	finite element method	STFT	short-time Fourier transform
FRF	frequency response function	S-N	stress-number of cycles
FFT	fast Fourier transform	VMD	variational mode decomposition
*H*(*Ω*)	the acceleration-stress FRF	σ(Ω)	frequency domain signal of simulation stress
$H^{R}(\Omega )$	the real part of *H*(*Ω*)	σ(Ν)	time domain signal of simulation stress
